# Is Fluoride the Culprit? Revisiting Evidence on Environmental Origins of Chronic Kidney Disease of Uncertain Etiology (CKDu): A Narrative Review

**DOI:** 10.3390/toxics13110966

**Published:** 2025-11-10

**Authors:** T. D. K. S. C. Gunasekara, P. Mangala C. S. De Silva, W. M. P. A. Wijesundara, G. G. T. Chaminda, Chula Herath, Sisira Siribaddana, Mercedes A. Bravo, Nishad Jayasundara

**Affiliations:** 1 Department of Zoology, Faculty of Science, University of Ruhuna, Matara 81000, Sri Lanka; gunasekara.sc166@fgs.ruh.ac.lk (T.D.K.S.C.G.); anjali.wijesundara97@gmail.com (W.M.P.A.W.); 2 Duke Global Health Institute, Duke University, Durham, NC 27708, USA; mercedes.bravo@duke.edu (M.A.B.); nj58@duke.edu (N.J.); 3 Department of Civil & Environmental Engineering, Faculty of Engineering, University of Ruhuna, Galle 80000, Sri Lanka; tusharac@cee.ruh.ac.lk; 4 Department of Nephrology, Sri Jayewardenepura General Hospital, Colombo 10100, Sri Lanka; chulaherath@gmail.com; 5 Department of Medicine, Faculty of Medical & Allied Sciences, Rajarata University, Saliyapura 50008, Sri Lanka; sisira.siribaddana@gmail.com; 6 Nicholas School of the Environment, Duke University, Durham, NC 27708, USA

**Keywords:** chronic kidney disease, drinking water, fluoride exposure, kidney injury

## Abstract

Fluoride is increasingly discussed as a geogenic risk factor for chronic kidney disease of uncertain etiology (CKDu); an epidemic of kidney disease is affecting hot tropical farming communities worldwide. Emerging evidence continues to support the association between high-fluoride exposure and kidney injury, particularly in regions with high fluoride levels. However, while fluoride’s geogenic nature leads to prolonged exposure through water and food sources, the direct impact on kidney health remains incompletely understood. This review explores the relationship between fluoride exposure and adverse kidney health outcomes, especially in the context of CKDu, synthesizing findings from epidemiological studies conducted worldwide. While a broad range of studies show widespread dental fluorosis prevalence in regions with high environmental fluoride levels in Sri Lanka, India, China, and Mexico, such correlation was not evident for CKDu and environmental fluoride levels. Notably, the spatial distribution patterns of CKDu and exposure risk through high fluoride levels in drinking water exhibit some inconsistencies, suggesting fluoride alone may not be the sole driver of CKDu. This review underscores the kidney health risks of fluoride exposure while emphasizing the need for further studies that consider multiple interacting factors beyond fluoride exposure in examination of environmental triggers of CKDu.

## 1. Introduction

Fluoride is a ubiquitous compound with sources ranging from drinking water to industrial processes, dental products, and certain food items. Communities worldwide are exposed to varying degrees of fluoride due to geographical, industrial, and lifestyle factors, thereby necessitating a comprehensive examination of its potential impact on health.

Fluoride exposure can occur through multiple routes. One of the primary sources of human exposure to fluoride is through drinking water. Fluoride levels in drinking water and other sources exhibit pronounced regional variations. Geological factors, such as the composition of underlying rocks and soil, play a crucial role in determining natural fluoride levels. Furthermore, anthropogenic activities and industrialization contribute to local variations in fluoride exposure [1]. Populations residing in regions with naturally occurring high fluoride levels in groundwater may experience elevated exposure, while others may encounter artificially increased levels due to fluoridation practices [2]. Industrial activities contribute to fluoride exposure through emissions and discharge. Certain industrial processes, such as phosphate fertilizer production and aluminum smelting, release fluoride into the surrounding environment. Proximity to such industries can result in heightened fluoride exposure for nearby communities [3]. Dental products, including toothpaste and mouthwash, are also common sources of fluoride for individuals. While these products are essential for oral health, they contribute to systemic fluoride exposure, especially in cases of improper use or ingestion by children [4]. Food items, both natural and processed, can contain varying levels of fluoride [5]. Certain crops accumulate fluoride from the soil [6], and the consumption of tea [7], and certain seafood [8] can contribute significantly to dietary fluoride intake. Additionally, the use of fluoride-containing pesticides may further contribute to fluoride levels in food [9].

Beneficial effects of low fluoride exposure are often discussed referring to dental health. For teeth and bone development and the prevention of dental caries, a fluoride concentration of ~0.7 ppm in drinking water is considered beneficial [10]. On the contrary, drinking-water fluoride levels above 1.5 ppm are associated with adverse health effects on the skeleton including skeletal fluorosis, osteoporosis, and arthritis [11]. Moreover, emerging research indicates that fluoride toxicity extends beyond calcified tissues, affecting vascular, nervous, reproductive, and urinary systems, and may contribute to renal injury in susceptible populations [12]. In this context, fluoride exposure has gained increasing attention as a potential risk factor of chronic kidney disease of uncertain etiology (CKDu).

CKDu is an emerging form of chronic kidney disease (CKD) characterized by progressive renal impairment in the absence of traditional risk factors such as diabetes mellitus, hypertension, or glomerulonephritis [13]. CKDu predominantly affects otherwise healthy adults, particularly in agricultural communities of tropical and subtropical regions, with a notable prevalence in Sri Lanka, Central America, and parts of India [14]. Clinically, the disease is often asymptomatic in its early stages and is typically non-proteinuric, making early detection challenging [15]. Histopathological findings commonly reveal tubulointerstitial damage rather than glomerular pathology, suggesting environmental, occupational, or multifactorial etiologies [16]. Epidemiological studies indicate associations with prolonged exposure to heat stress, dehydration, agrochemicals, heavy metals, and contaminated water, though no single causal factor has been definitively established. CKDu represents a significant public health concern due to its insidious onset, high morbidity, and socio-economic impact on affected communities, underscoring the need for multidisciplinary research to elucidate its pathophysiology, risk factors, and preventive strategies.

Building on these environmental and occupational exposures, fluoride at elevated levels has emerged as a potential contributor to CKDu in affected regions. Throughout the past few decades, a large number of studies have extensively investigated the adverse health outcomes associated with high-fluoride exposure on human health and in laboratory animal models. However, much of this work has focused on skeletal and dental outcomes, providing limited insight into renal pathophysiology and its relevance to CKDu. Laboratory studies with different animal models and in vitro studies have provided insights into potentially toxic effects of fluoride exposure. However, the doses of exposure in many of these studies are higher than environmentally relevant levels. As implied by these studies, exposure to elevated fluoride levels is supposed to elicit a number of detrimental health effects including structural and functional impairments in soft tissues, the gastrointestinal system, the brain, and the liver, along with cytotoxicity, neurotoxicity, hypersensitivity and immunotoxicity, genotoxicity, carcinogenicity in multiple organs, developmental effects, and disruptions in production and secretion of the thyroid hormones [12].

Given the rising concern over CKDu in fluoride-endemic regions, understanding how environmental fluoride exposure influences kidney structure and function is essential. Considering the multiple and ubiquitous sources of fluoride exposure, and the emerging evidence indicating a role for fluoride in chronic kidney disease outcomes, this review aims to contextualize fluoride exposure within the broader landscape of CKDu research. Specifically, we review (i) soil geochemistry and environmental fluoride, (ii) regulatory thresholds for fluoride exposure, (iii) health consequences of fluoride, and (iv) risks to kidney health, including associations of fluoride exposure with prevalence of CKDu. We seek to provide a nuanced understanding of the current state of knowledge, identify research gaps, and offer insights that may inform public health policies and future investigations. In doing so, we aim to contribute to the ongoing dialog surrounding CKDu and fluoride exposure, fostering a foundation for targeted interventions and improved health outcomes for affected populations.

## 2. Material and Methods

This narrative review was conducted to summarize current evidence on environmental fluoride exposure and its potential impact on kidney health, with a particular focus on tropical communities affected by chronic kidney disease of uncertain etiology (CKDu). Relevant literature was identified through comprehensive searches of PubMed, Scopus, and Web of Science databases. The search terms included “fluoride” or “fluorosis”, “kidney” or “renal injury” or “nephrotoxicity”, “chronic kidney disease” or “CKDu” or “endemic nephropathy”, and “groundwater” or “drinking water” or “environmental exposure”. In addition, references of key articles and relevant review papers were also reviewed to identify additional sources.

Articles published in English between 2000 and 2024 were considered. Studies were included if they were peer-reviewed original research reporting environmental or dietary fluoride exposure in humans or relevant animal models, assessed kidney outcomes (including renal biomarkers, function, histopathology, or epidemiology), and were conducted in regions where CKDu has been reported or where groundwater fluoride exposure is high. Studies were excluded if they focused solely on dental or skeletal fluorosis without kidney outcomes, were narrative commentaries, conference abstracts, or case reports without original data, or were conducted exclusively in populations with conventional CKD risk factors such as diabetes or hypertension.

Titles and abstracts of identified articles were screened for relevance, and full texts of potentially eligible studies were reviewed. Selected studies were synthesized qualitatively to provide a narrative overview of current knowledge, highlight research gaps, and suggest directions for future investigations.

## 3. Evidence Synthesis

### 3.1. Fluoride Exposure Through Drinking Water: A Global Perspective

Human exposure to fluoride primarily occurs through drinking water. According to the World Health Organization (WHO), the occurrence of dental fluorosis is more likely when the drinking water fluoride content exceeds 1.5 ppm, whereas skeletal fluorosis is more likely when drinking water fluoride content exceeds 3 ppm. As a protective measure against these health impacts, WHO recommends drinking-water fluoride levels below 1.5 ppm [17].

**Figure 1:**
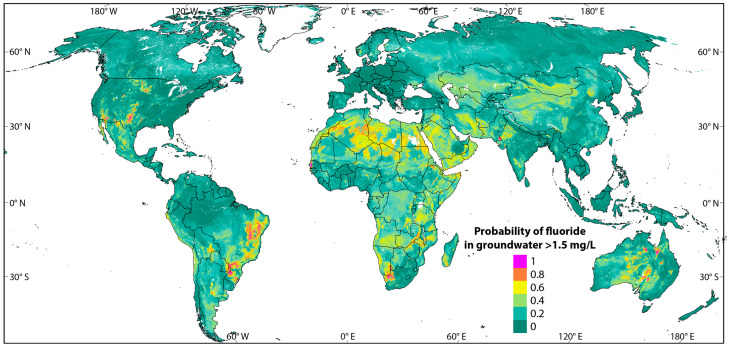
Probability of the likelihood of elevated fluoride contents in drinking water above the WHO recommendations in global perspective. Adapted from Podgorski and Berg, 2022 [18]. Online source: GAP, Groundwater Assessment Platform (2015), Swiss Fedral Institute of Aquatic Science and Technology (Eawag), Postfach 611, 8600 Dübendorf, Switzerland. https://www.gapmaps.org/Home/Public#, accessed on 31 October 2025.

Adhering to the WHO recommendation for drinking water fluoride content (1.5 ppm), the probability of high-fluoride exposure in global communities is illustrated in Figure 1 as per the predictive analysis by Podgorski and Berg, 2022 [18].

**Figure 2:**
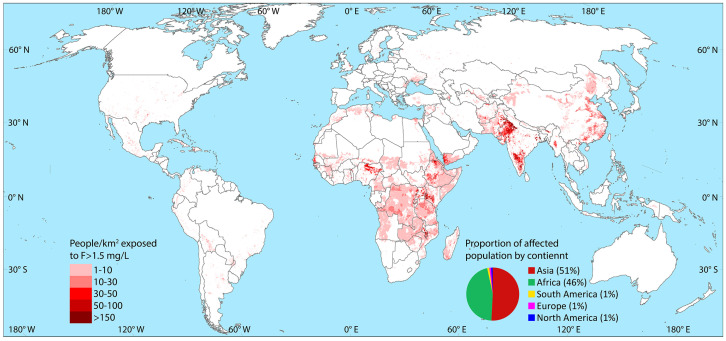
Estimated population potentially exposed to fluoride concentrations in drinking water greater than 1.5 ppm (WHO recommendation). Adapted from Podgorski and Berg, 2022 [18]. Online source: GAP, Groundwater Assessment Platform (2015), Swiss Fedral Institute of Aquatic Science and Technology (Eawag), Postfach 611, 8600 Dübendorf, Switzerland. https://www.gapmaps.org/Home/Public#, accessed on 31 October 2025.

According to the estimations of Podgorski and Berg, 2022, the population exposed to drinking water with fluoride concentrations above 1.5 ppm is represented in Figure 2 [18]. This estimation is based on random forest modeling for areas with a greater than 25% probability of incurring high fluoride in groundwater by multiplying the total population by the hazard percentage and the proportion of domestic water usage coming from untreated groundwater [18].

As implied by this recent estimation, hotspots of high risk of elevated fluoride exposure include central Australia, western North America, eastern Brazil, Africa, and multiple areas in Asia including India and Sri Lanka. Out of the total global population, 180 million people are likely to be exposed to high fluoride through drinking water, with a majority residing in Asia (51–59%) and Africa (37–46%) [18].

In addition to drinking water alone, fluoride exposure is also identified through beverages and food as evidenced by multiple studies. Analysis of fluoride contents in sugary beverages consumed by children in several Mexican states has reported the presence of fluoride above the regulations, particularly, cheap beverages. According to risk assessment, the researchers identified a potential risk of dental fluorosis development for the children who daily consume these beverages having fluoride contents above 0.7 ppm [19]. Similar risk of high fluoride exposure through soft drinks and tea have been reported from multiple countries including Poland [20,21], Ethiopia [22], Tibet [23]. Importantly, the contents of fluoride in food, milk, tea and other beverages depends on soil geochemistry, and the risk of high fluoride exposure remains high in regions with high geogenic fluoride contents such as the Rift Valley in Ethiopia which is a region with endemic fluorosis [24,25].

### 3.2. High-Fluoride Exposure and Associated Kidney Health Risks

Understanding the kidney health implications of chronic fluoride exposure is critical, as the kidneys are the primary route of fluoride excretion and thus highly susceptible to its toxic effects. Once absorbed through the gastrointestinal tract, fluoride rapidly enters systemic circulation, with a fraction deposited in bones and teeth while most is eliminated via urine. Fluoride stored in skeletal tissues can also be remobilized into the bloodstream, sustaining a continuous renal burden. Consequently, persistent or excessive exposure may induce cumulative toxic effects on kidney function [12]. For example, fluoride can form complexes with essential metal ions such as calcium and magnesium, disrupting electrolyte balance and impairing renal tubular reabsorption processes. These disruptions may alter cellular metabolism, promote oxidative stress, and contribute to tubular and glomerular injury over time as discussed in the later sections.

Evidence from human urine measurements indicate the susceptibility of kidneys to fluoride. For example, a study conducted in Nicaragua among 60 healthy adults (aged > 20 years) residing in regions with high (3.05 ppm) and low (0.04 ppm) drinking-water fluoride levels demonstrated significant correlations between water fluoride concentration and fluoride levels in biological specimens. The estimated total daily fluoride intake showed strong correlations with the fluoride contents in urine (r = 0.730, *p* < 0.001), plasma (r = 0.729, *p* < 0.001), fasting whole saliva (r = 0.653, *p* < 0.001), and hair (r = 0.603, *p* < 0.001), with moderate correlations in fingernails (r = 0.502, *p* < 0.001) and toenails (r = 0.556, *p* < 0.001) [26]. These findings highlight urinary fluoride as a sensitive biomarker of environmental exposure and underscore the kidney’s central role in fluoride elimination and likely susceptibility to fluoride toxicity.

Mechanistically, fluoride can form complexes with essential metal ions such as calcium and magnesium, disrupting electrolyte balance and impairing renal tubular reabsorption processes. These disruptions may alter cellular metabolism, promote oxidative stress, and contribute to tubular and glomerular injury over time.

#### 3.2.1. Mechanisms of Fluoride-Mediated Kidney Injury

The kidneys are among the primary target organs of fluoride toxicity, where excessive exposure mainly through drinking water and diet can induce structural and functional alterations [27]. Fluoride toxicity at high exposure levels has been demonstrated in multiple animal studies as indicated by increased kidney injury markers in a dose-dependent manner [28]. For instance, a study by Cárdenas-González et al., 2013 identified significantly elevated urinary expression of several kidney injury markers including Kidney injury molecule (KIM-1), clusterin (CLU), osteopontin (OPN) and heat shock protein 72 (HSP-72), β-2-microglobulin, and cystatin-C in rats following exposure to fluoride-containing drinking water (50 ppm) [29]. Moreover, histopathological findings under Light microscopy evidenced a tendency towards a dose dependent increase of tubular damage [29]. Particularly, increased urinary KIM-1 levels have been observed in rats with high-fluoride exposure suggesting fluoride-induced kidney injury [30]. In addition, studies with several other animal models including zebra fish [31], mice [32], ducks [33], and rabbits [34], have demonstrated deformities in renal structure characterized by shrunken and lobulated glomeruli, dilated renal tubules, infiltration of inflammatory cells in renal tissues, and vacuolar degeneration of the tubular epithelium in response to high-fluoride exposure [12]. Moreover, high-fluoride exposure also accounts for structural alterations of proximal, distal, and collecting tubules [35].

Emerging evidence from in vivo and in vitro studies indicates that fluoride-induced kidney injury involves a complex interplay of cellular and molecular mechanisms, with potential relevance to the pathogenesis of CKD. Chronic fluoride exposure at elevated levels can lead to certain histopathological changes in renal tissues, including tubular degeneration, glomerular atrophy, and interstitial fibrosis. Such structural alterations in kidneys compromise the renal performance, potentially leading to declined renal function over time [36].

**Figure 3:**
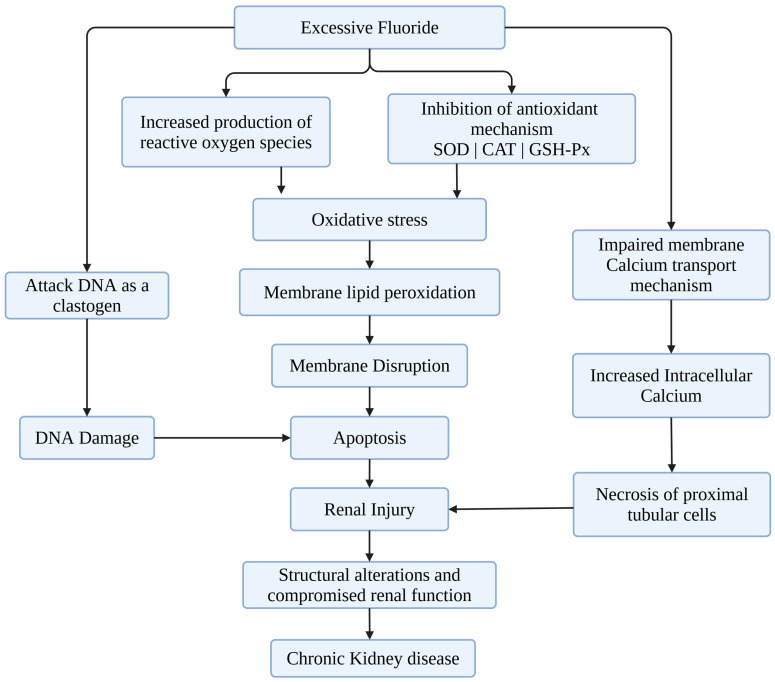
An overview of the mechanisms of excessive fluoride–mediated nephrotoxicity based on the findings from in vivo and in vitro studies.

Fluoride enhances the generation of reactive oxygen species (ROS), depletes endogenous antioxidants such as superoxide dismutase (SOD), catalase, and glutathione, leading to oxidative stress. ROS results in cell injury by damaging lipids, protein, and DNA when they are produced in excess or the antioxidant defense is impaired. Oxidative stress affects regular cellular function and triggers inflammatory responses, further supporting renal injury. Moreover, fluoride-mediated nephrotoxic effects also encompass inhibition of crucial enzyme activities that are necessary for renal function. Particularly, the enzymes involved in energy metabolism such as ATPases are impacted, leading to diminished energy production and compromised cellular functions. In addition, fluoride exposure disrupts the electrolyte balance which is crucial for maintaining cellular homeostasis and optimal renal function. Chronic exposure to elevated fluoride levels has been linked to alterations in the expression of apoptosis-related genes, resulting in excessive renal cell death, loss of functional nephrons, and progressive decline in kidney efficiency. In addition, fluoride has been implicated in the development of renal fibrosis, marked by abnormal accumulation of extracellular matrix components. This fibrotic remodeling increases tissue rigidity, hampers glomerular filtration, and may ultimately progress to chronic kidney disease [36]. Prolonged exposure to elevated fluoride levels can overwhelm renal clearance mechanisms, leading to the accumulation of fluoride within kidney tissues and consequent toxicity [37]. A summary of fluoride-mediated nephrotoxicity pathways is given in Figure 3 [38,39].

However, the above mechanisms of high-fluoride-exposure-related renal injury are mostly based on animal and laboratory investigations. In most of the studies that demonstrate toxicity effects of fluoride, animals or cells are exposed to higher concentrations of sodium fluoride. For example, administering fluoride at higher doses (12 mg/kg, 24 mg/kg, and 48 mg/kg) for 43 days showed oxidative stress in renal tissues as characterized by increased levels of reactive oxygen species (ROS) and declined abilities of anti-superoxide anion (ASA) and anti-hydroxyl radical (AHR), glutathione (GSH) content, as well as the activities and mRNA expression levels of superoxide dismutase (SOD), catalase (CAT), glutathione reductase (GR), and glutathione peroxidase (GSH-Px). Moreover, ingestion of fluoride resulted in significant elevations in serum creatinine (SCr), serum uric acid (UA), blood urea nitrogen (BUN), and the activities of urinary N-acetyl-b-D-glucosaminidase (NAG), renal lactate dehydrogenase (LDH), and reduced activities of sodium–potassium adenosine triphosphatase (Na^+^/K^+^-ATPase) and acid phosphatase (ACP) in the kidney [40]. Similarly, a significant decline in antioxidant activity in renal tissues has been reported in rat models, resulting from drinking sodium-fluoride-containing water: 50 ppm of fluoride for 60 days [41], and 600 ppm for 1 week [42].

Moreover, a study with a rat model reported a significant increase in serum and urinary creatinine, urea, uric acid, and KIM-1 following exposure to drinking-water fluoride concentrations of 50 and 100 ppm for 6 months. Histopathological examination of the kidney revealed focal interstitial nephritis (FIN) along with moderate to severe tubular degeneration, mild or low-grade inflammatory infiltration, and moderate tubular dilation [43]. In another study, rats received fluoride-containing water at three concentrations, 0.5 ppm, 5 ppm, and 20 ppm. Significantly elevated serum creatinine levels were observed only in the highest exposure (20 ppm) group. Also, renal histopathology examinations identified no structural abnormalities in any of the three groups [44]. On the contrary, administration of higher fluoride contents, 50 ppm, 100 ppm, and 200 ppm via drinking water for 120 days resulted increased apoptosis and DNA damage in renal tissues of rats [45].

However, it is important to recognize that the fluoride concentrations used in these studies are substantially higher than those found in natural groundwater or drinking water sources, whereas fluoride concentrations in most natural water sources are typically within 0.5–5 mg/L. These experimental doses therefore represent acute or sub-chronic exposure levels that are several orders of magnitude greater than environmentally relevant concentrations. Consequently, while such studies provide valuable mechanistic insights into fluoride toxicity, they may not directly reflect the pathophysiological processes occurring under chronic, low-dose exposure conditions.

In this context, emerging findings also suggest that fluoride may interact with other environmental factors such as water hardness, trace metals, and agrochemicals to potentiate renal injury through synergistic oxidative and inflammatory mechanisms. Fluoride nephrotoxicity may be amplified by concurrent exposure to water hardness (calcium and magnesium ions), heavy metals (such as cadmium and arsenic), and agrochemical residues. These co-exposures can modify fluoride bioavailability, enhance oxidative and inflammatory stress, and intensify tubular injury. For example, high water hardness may facilitate the formation of insoluble fluoride complexes, altering renal handling of calcium and magnesium, while trace metals can potentiate ROS generation and inhibit antioxidant defenses [46]. Together, these synergistic mechanisms suggest that chronic exposure to fluoride, particularly in conjunction with other environmental stressors prevalent in CKDu-endemic regions, may create a cumulative nephrotoxic milieu that underlies the disease’s multifactorial etiology.

#### 3.2.2. Evidence from Community Studies

A growing body of research has investigated the relationship between elevated fluoride levels in drinking water and adverse kidney health outcomes within various communities worldwide. This section presents an overview of key studies conducted in India, Mexico, and China as examples to highlight kidney health implications of high fluoride exposure.

In India, due to higher fluoride contents in ground water, 21 states out of 35 states are known to have endemic fluorosis [47,48]. Relatively higher fluoride exposure levels have been identified in children and adults in multiple communities across different regions in India along with evidence of dental fluorosis. A case–control study with 824 school children (8–15 years of age) from 10 schools in the Doda district of Jammu and Kashmir states in India revealed a significant risk of kidney injury with high fluoride exposure. These researchers selected 8 schools from high fluoride regions with evidence of dental fluorosis where drinking-water fluoride content ranged between 1.43 and 3.84 ppm, along with a control group representing 2 schools in low fluoride regions (drinking-water fluoride content below 1 ppm). Dental fluorosis was identified in different grades in more than 95% of the affected regions. Urinary fluoride level of affected group (3.28 ± 1.71 ppm) was significantly higher compared to the control group (1.91 ± 0.64 ppm). The mean serum creatinine level (0.85 ± 0.35 mg/dL) of the affected children was significantly high compared to the control (0.45 ± 0.16 mg/dL). Moreover, mean eGFR of the affected children (84.1 ± 33.1 mL/min/1.73 m^2^) was significantly lower compared to the control (120.9 ± 27.4 mL/min/1.73 m^2^). These findings suggest a link between high-fluoride exposure and potential kidney injury in children [49].

A biopsy study with Indian children with nephrotic syndrome lead characterization of microscopic evidence of kidney injury linked with high fluoride exposure. The participants represented three groups: the high-exposure patient group (urine fluoride level 4.01 ± 1.83 ppm), the normal-fluoride patient group (0.61 ± 0.17 ppm), and the healthy control group (urinary fluoride level 0.56 ± 0.15 ppm). Compared to the normal-fluoride group, apoptosis was observed at an increased level in the high-fluoride-excretion group. Furthermore, tubular epithelial damage associated with high-fluoride exposure was observed in multiple forms including cell swelling and lysis, cytoplasmic vacuolation, nuclear condensation, apoptosis, and necrosis [50]. However, the children with high urinary fluoride levels had previously been diagnosed with nephrotic syndrome; therefore, the observed renal damage cannot be attributed solely to fluoride exposure, as nephrotic syndrome itself can cause renal injury.

Another study with 842 young children in three Indian villages identified notably higher incidence of dental fluorosis: 51.90% in Jhajjar, 94.63% in Dadanpur and 36.84% in Dariyapur. Significant, positive correlations of fluorosis with serum and urinary fluoride levels, and drinking-water fluoride levels were observed [51]. A similar study with 136 school children from two villages (Gobindanagar and Bhagabandh) in the Purulia District of West Bengal in India, identified dental fluorosis in 36.9% of children within the age range 12–14 years. Urinary fluoride ranges in children with dental fluorosis in Gobindanagar and Bhagabandh were 0.08–6.7 ppm and 1.5–7.9 ppm, respectively. However, none of these studies assessed the kidney health of the participants [52].

In many regions of Mexico, higher fluoride exposures with evidence of dental fluorosis and adverse kidney health outcomes in adults and children are reported. A cross-sectional study with 239 adult residents from three municipalities in Chihuahua, México, reported potential associations of fluoride exposure with kidney injury. The mean fluoride contents in drinking water and urine were 1.5 ppm (0.19–1.8 ppm) and 2.0 ppm (1.1–3.5 ppm) ppm, respectively. Nearly 51% of samples reported fluoride levels above 1.5 ppm. Moderate and severe dental fluorosis was observed in 18.7% of participants. Multiple linear regression models identified a positive association of urinary fluoride level with multiple urinary markers including albumin (β = 0.56, *p* < 0.001), cystatin-C (β = 0.022, *p* = 0.001), KIM-1 (β = 0.048, *p* = 0.008), osteopontin (β = 0.38, *p* = 0.041), and eGFR (β = 0.49, *p* = 0.03) [53].

A similar cross-sectional study with 374 school children from the municipalities of Hidalgo del Parral and Aldama in Chihuahua, México, reported supportive findings to the above observations. In the region with high F exposure, the mean (IQR) fluoride levels in drinking water and urine were 1.93 ppm (0.3–2.1 ppm) and 2.7 ppm (2.0–3.6 ppm), respectively, with a prevalence of 41.7% for dental fluorosis. In multiple linear regression models, urinary F was positively associated with eGFR (β = 1.3, *p* = 0.015), urinary cystatin-C (β = −8.5, *p* = 0.043), VCAM-1 (β = 111.1, *p* = 0.019), ICAM-1 (β = 57, *p* = 0.032), and cIMT (β = 0.01, *p* = 0.032). An inverse association was observed with urinary cystatin-C (β = −8.5, *p* = 0.043) and serum cystatin-C (β = −9.6, *p* = 0.021), and no significant associations with ET-1 (β = 0.069, *p* = 0.074) and KIM-1 (β = 29.1, *p* = 0.212) were found [54]. A study with 1742 adolescents also reported substantial associations of fluoride with renal function markers. The mean (standard error) fluoride contents in drinking water and plasma were 0.48 (0.03) ppm and 0.40 (0.01) μmol/L, respectively. Plasma fluoride was significantly associated with eGFR (β = −10.36, *p* = 0.01), and BUN (β = −1.29, *p* < 0.001) [55].

Multiple regions in China and India have also been identified with higher geogenic fluoride contents with adverse health impacts on residential communities. A cross-sectional study with 1070 adults (mean ± SD age was 58.21 ± 10.87 years) representing four villages in Wenshui County, Shanxi Province in China investigated kidney health outcomes linked with fluoride exposure using urinary NAG, serum retinol binding protein (RBP), serum urea, serum C3, serum uric acid, and serum α1-microglobulin. The mean urinary fluoride concentration was 1.62 ppm. Positive correlations of urinary fluoride concentrations were identified with both urinary NAG and serum urea. In adjusted regression models, a 1 ppm increment of urinary fluoride was associated with a 1.583 U/L increase in urinary NAG and a 0.199 mmol/L increase in serum urea [56]. A similar study with 320 (two age groups: 21–40 years and 41–60 years) adults residing in fluoride-endemic villages (mean fluoride range in drinking water ranged 1.5 ppm to 4.1 ppm) of the YSR Kadapa district in India identified a strong positive correlation of urinary fluoride levels with serum creatinine (*p* = 0.005), and blood urea nitrogen (*p* = 0.045). Moreover, drinking-water fluoride levels also showed positive correlation with serum creatinine (*p* = 0.0019) and BUN (0.042). However, these renal markers showed no significant difference between the subjects with fluorosis and without fluorosis in both age groups. Irrespective of fluorosis, these renal markers significantly differed between the two age groups [57].

While above community studies suggest potential positive associations of urinary or plasma fluoride levels with renal biomarkers, the findings appear to be inconsistent. For instance, KIM-1, which is a sensitive indicator of kidney injury, has been reported to show a positive association with fluoride exposure in some studies, whereas other studies have found weak or null associations between urinary or plasma fluoride levels and KIM-1. Furthermore, while several studies reported associations between fluoride levels and conventional renal markers such as serum creatinine and eGFR, others found no significant relationships. Differences in biomarker sensitivity, exposure assessment methods, and co-exposure to nephrotoxicants such as cadmium or arsenic may partly explain these discrepancies. For instance, urinary KIM-1, an early marker of tubular injury, was elevated in some Mexican cohorts but not in others with comparable fluoride exposure. Variations in study design, water hardness, hydration status, and population characteristics may further influence biomarker responses. Overall, these mixed findings highlight the need for standardized biomarker panels and longitudinal studies to clarify the causal links between fluoride exposure and kidney injury.

Moreover, as discussed previously, although urinary fluoride levels showed strong positive association with serum creatinine and blood urea nitrogen, there was no significant elevation of these markers in subjects with fluorosis, whereas the presence of fluorosis is an indication of chronic high-fluoride exposure (Table S1). Thus, associations of urinary, plasma, or drinking-water fluoride contents with renal injury biomarkers, alone may not provide strong evidence to establish a solid link between high-fluoride exposure and kidney injury at the normal environmental exposure settings.

#### 3.2.3. Fluoride and CKDu

**Figure 4:**
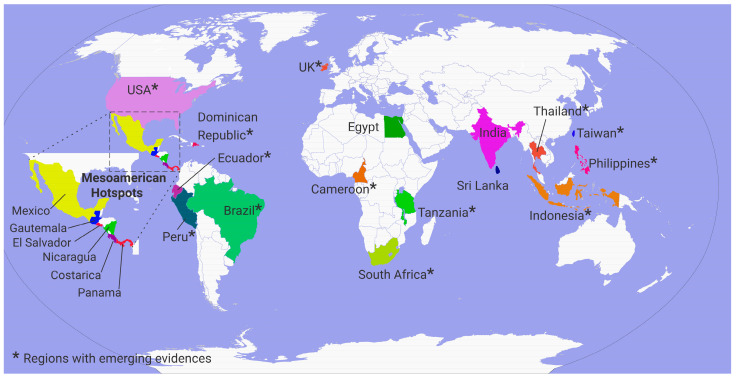
Global prevalence of CKDu: currently identified hotspots and regions with emerging evidence for the presence of CKDu adapted from Gunasekara et al., 2020 [58].

Chronic Kidney Disease of Uncertain Etiology (CKDu) presents a complex health challenge, affecting communities particularly in tropical agricultural settings. This nephropathy is characterized by a progressive decline in kidney function without definitive causative factors and devoid of traditional risk factors of chronic kidney disease such as diabetes, hypertension, and aging. Over the past two decades, CKDu has emerged as a global concern, with reports from multiple tropical and subtropical regions suggesting that this condition is not confined to a single country or continent. Mapping these occurrences provides valuable insight into its widespread yet regionally clustered nature. CKDu has manifested in hotspots across the globe, from the rice paddies of Sri Lanka to the sugarcane fields of Central America posing a substantial challenge to healthcare systems worldwide. The prevalence of CKDu exhibits notable geographical heterogeneity, with hotspots identified in Sri Lanka, Mesoamerica, and parts of India, as illustrated in Figure 4.

Interestingly, the confined nature of observed geographical and socio-economic CKDu disease patterns implies geo-environmental and/or occupational risk factors [59]. Prolonged exposure to agrochemicals—including glyphosate formulations and other nephrotoxic pesticides—has been hypothesized to contribute to tubular damage or to mobilize heavy metals from soil into groundwater [46,60,61,62,63,64]. Furthermore, long-term consumption of untreated groundwater with nephrotoxic contaminants been associated with renal stress leading to injury and progressive deterioration of renal function [62,65,66,67]. Recurrent heat stress and dehydration, particularly among outdoor agricultural workers, are believed to induce repeated subclinical kidney injury, leading to declined renal function over time [68,69]. Together, these factors underscore the multifactorial and context-dependent nature of CKDu, where environmental, occupational, and lifestyle exposures likely act in combination to influence disease onset and progression. Importantly, some of these factors may interact with fluoride in the environment and within an individual, amplifying or mitigating the impact of fluoride exposure on CKDu incidence. Understanding the regional nuances is pivotal for developing targeted interventions and preventive measures tailored to the specific challenges faced by each community.

The potential link between fluoride and CKDu has gained prominence in recent years with studies from diverse regions providing evidence. Fluoride exposure through drinking water has been a subject of considerable concern regarding its impact on kidney health in various regions, with many studies based in Sri Lanka. The subsequent section summarizes multiple studies, aiming to contextualize the potential association between fluoride exposure and CKDu.

##### Evidence from Epidemiological and Hydrological Studies

Understanding the potential link between environmental fluoride exposure and renal health outcomes requires careful examination of community-based evidence from diverse geographic regions. Epidemiological investigations have reported varying associations between fluoride exposure and markers of kidney function, but these findings must be interpreted cautiously. Differences in study design, exposure assessment, co-exposures, and population characteristics, as well as the possibility of reverse causality, where impaired kidney function itself may influence fluoride retention can complicate causal inference.

A case–control study of 116 biopsy-confirmed non-dialysis CKDu patients and 77 age-matched controls in two CKDu-impacted regions in Sri Lanka (Girandurukotte and Wilgamuwa) found that CKDu patients had significantly higher serum and urinary fluoride levels than controls. In CKDu patients, mean serum and urine fluoride levels were 1.39 ± 1.1 ppm (range: 0.47–9.58 ppm) and 1.53 ± 0.8 ppm (range: 0.45–6.92 ppm), compared to 1.07 ± 0.3 ppm (range: 0.51–1.92 ppm) and 1.26 ± 0.6 ppm (range: 0.36–3.8 ppm) in controls, respectively. Urinary fluoride levels also varied significantly with CKD stage in CKDu patients [70]. More consistent findings to these were also identified by Nanayakkara et al., in their study with 311 biopsy-confirmed CKDu patients and 276 controls from Girandurukotte and Medawachchiya. Mean serum fluoride levels of the patients ranged between 0.035 ppm and 0.124 ppm, and serum fluoride levels increased with decreasing eGFR. Analysis of drinking-water samples identified fluoride contents below the WHO recommended level (1.5 ppm) in 95% of the samples, suggesting little evidence for fluoride exposure through drinking water in the area [71]. Referring to their study in North Central Province, Wasana et al., reported contrasting findings. Accordingly, fluoride levels in drinking-water samples collected from CKDu-impacted communities remained above the WHO recommended limits. Furthermore, levels of Cd, As, and Al in water were found comparable across all study regions and remained below WHO permitted levels (3 ppb for Cd and 10 ppb for As). Although a positive correlation of drinking-water fluoride concentration with the prevalence of CKDu was identified, the prevalence of CKDu was low in certain regions with high fluoride contents [66]. Consistent findings were reported by Perera et al., in a similar study to evaluate the contamination status of heavy metals/metalloids of the drinking water and agricultural soil in two grama niladhari divisions in CKDu-endemic areas (Eppawala and Ambagaswewa) in the North Central Province in comparison to a control GND (Dambethalawa) in Ampara district in Sri Lanka. Mean drinking-water fluoride levels in CKDu endemic GNDs: Eppawala (1.87 ppm; range: 0.09–3.98 ppm) and Ambagaswewa (1.72 ppm; range: 0.05–4.00 ppm) were markedly high compared to the control area (0.40 ppm; range: 0.10–1.10 ppm). According to the analysis, the concentrations of Cd, As, Pb, Cr, Cu, and Zn in drinking-water samples collected from both CKDu-endemic regions remained below the recommended thresholds [72]. Moneragala district is also a region with emerging evidence of CKDu located in southern Sri Lanka. Based on the screening of 46,754 individuals in the Monaragala district, Liyanage et al. estimated the prevalence of CKDu as 6.7%. Significantly higher levels of fluoride were identified in well water consumed by CKDu patients compared to the wells utilized by healthy individuals. Fluoride levels above the recommended threshold (1 ppm) were reported in 87% of the wells utilized by CKDu patients. Furthermore, significantly higher concentrations of multiple metal ions (Na^+^, K^+^, Mg^2+^, Ca^2+^, Sr^2+^, Ba^2+^) were identified in well water consumed by CKDu patients, However, the concentrations of As, Pb, and Cd remained at permissible limits in all wells [73]. Collectively these studies highlight significant elevation of urinary and serum fluoride excretion, presence of significantly higher fluoride contents and hardness in well water consumed by CKDu patients, and the presence of nephrotoxic ions in low levels. However, the correlations between elevated fluoride levels and CKDu shown in these studies are not fully convincing regarding the causality of CKDu, as reduced renal clearance in CKDu patients could itself elevate serum and urinary fluoride, suggesting the possibility of reverse causation.

**Figure 5:**
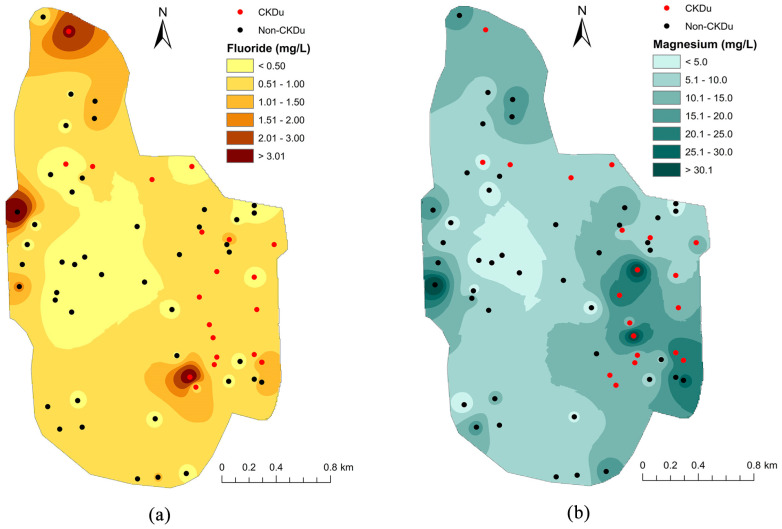
Spatial distribution of (**a**) groundwater fluoride, and (**b**) magnesium in Ginnoruwa area in relation to CKDu incidence. Adapted from Balasooriya et al., 2019 [75].

Importantly, apart from drinking-water fluoride, multiple studies in CKDu-endemic and non-endemic regions in Sri Lanka have reported mixed findings on nephrotoxic ion levels in drinking-water sources. For instance, a study in Wewelketiya (a CKDu-endemic region in North Central Province) identified Cd and Pb above recommended limits in 60% and 40% of samples, respectively, but fluoride exceeded permissible limits in 80% of wells. The coexistence of multiple potential nephrotoxicants complicates attribution of CKDu etiology to fluoride alone [74]. In 2019, Balasooriya et al. conducted investigations on fluoride and hardness in relation to CKDu prevalence in the Ginnoruwa area, which is a known CKDu hotspot in Sri Lanka [75]. The analysis identified significantly higher levels of total hardness, electrical conductivity, and several ions (Ca^2+^, Mg^2+^, F^−^, Cl^−^, PO_4_^3−^, SO_4_^2−^) in wells used by CKDu patients compared to the nearby wells utilized by healthy controls. The presence of As, Cd, and Pb showed no significant difference between wells while the concentrations of these ions remained below the WHO recommended limits [75]. A detailed elaboration on the geospatial distribution of groundwater fluoride and magnesium in the study region is given in Figure 5 [75].

According to the interpretations of this study, researchers suggest that drinking-water fluoride and hardness may act synergistically contributing to kidney injury and progression of kidney damage [75]. As it appears in the ground water fluoride distribution map (Figure 5a), a majority of the wells of CKDu patients are located in areas with drinking-water fluoride contents below 1.5 ppm, along with a few wells located in high fluoride areas. However, there are some wells utilized by healthy subjects that remain at very close proximity to the wells utilized by CKDu patients with more consistent fluoride and magnesium contents. Hence, to gain more precise insights into the association of fluoride and hardness, careful consideration of the other exposures and lifestyle practices of the participants are required. A similar hydrological investigation was conducted by Botheju et al. in Girandurukotte (a CKDu-impacted region in the Badulla District) Grama Niladhari division (GND) along with a control area, Dambethalawa GND (CKDu non-prevalent region in the Ampara district) in Sri Lanka [76]. Elevated fluoride levels above the recommended thresholds were observed in 81% of water samples in CKDu-affected regions (range: 0.244–5.743 ppm), while this percentage was 28% in CKDu non-affected regions. A higher degree of water hardness was identified in CKDu-impacted regions (22.98–174.01 ppm), while soft water was identified in the control region. Moreover, analysis of water samples revealed significantly higher concentrations of nephrotoxic ions Cd (9.78–187.25 ppb), Pb (0.08–0.66 ppb), As (20.76–103.30 ppb), and Cr (0.03–0.34 ppm). According to Botheju et al. prolonged exposure to nephrotoxic ions, high fluoride, and hardness may contribute to CKDu in affected regions [76]. However, this study has not incorporated the residencies of CKDu patients along with the geospatial map of fluoride, hardness, and nephrotoxic ion concentrations in Girandurukotte GND.

Furthermore, findings from multiple studies report coexistence of nephrotoxic ions and hardness with fluoride in drinking-water samples in CKDu-impacted regions. In particular, distinct dry and wet seasons are identified in CKDu-impacted regions, and variations in the concentrations of the contaminants are also likely to alter the exposure risks. A comparative survey on water quality in the Thunukkai Divisional Secretariat (a CKDu-impacted region in Mullaitivu District in Sri Lanka) also identified elevated levels of fluoride (mean: 1.73 ppm, range: 22.3–0.1 ppm) and hardness (mean: 295.76 ppm, range: 683–39 ppm) in drinking-water samples [77]. Moreover, exceeding the established standards in Sri Lanka, the water samples reported elevated levels of fluoride (39.5%), hardness (65.4%), arsenic (23.4%), chloride (31.42%), and nitrate (15.2%), when taken as a percentage of the total number of water samples collected, arsenic content and total hardness showed positive correlations with serum creatinine in CKDu patients in the area [77]. Similarly, an analysis of water samples in 34 wells (27 dug wells and 7 tube wells) in Anuradhapura in Sri Lanka district identified elevated total dissolved solid and magnesium levels exceeding the maximum allowable limits exclusively in the high risk areas of CKDu showing significant associations of water hardness and ionicity in groundwater with the incidence of CKDu [78]. Moreover, a separate study with 60 water samples in Anuradhapura district found that water quality parameters including alkalinity, TDS, hardness, and magnesium were not significantly affected by the seasonal variations in rainfall in the area, leading to their presence at higher levels in well water throughout the year [79].

The Uva province is another region in Sri Lanka where CKDu is reported among farming communities in Sri Lanka. A recent study with 260 groundwater samples, identified a low fluoride level (mean <1 ppm) with relatively low hardness (mean ± SD: 234 ± 170 ppm), compared to the levels reported in the North Central Province. However, 67% of the dug wells and 59.7% of the tube wells reported fluoride contents above the threshold of 1.5 ppm in water showing a likelihood of high fluoride exposures within the communities. In logistic regression models, fluoride and phosphate in ground water had significant associations with the progression of CKDu among the inhabitants [80]. In addition, a number of hydrological investigations in CKDu-impacted regions in Sri Lanka have evidenced the presence of fluoride, hardness, and a number of cations and anions at elevated levels compared to the drinking-water samples in CKDu non-prevalent regions in the country [59,64,65,81,82,83,84]. Considering the evidence reported in these hydrological investigations, the presence of such ions at elevated levels, exceeding the recommended levels, is discussed as a potential factor that contributes to kidney injury and CKDu in localized hotspots. Importantly, the synergetic impact of these ionic combinations in drinking water needs to be studied further to determine the mechanistic basis of kidney injury associated with these ions in relation to CKDu.

Apart from Sri Lanka, several studies have also investigated associations of high-fluoride exposure and kidney health. A study analyzed composite groundwater samples collected from 40 selected villages in Uddanam and 100 villages of the other nine districts in Andhra Pradesh, both in India [85]. Higher incidence of acidity in drinking water (pH < 6.5) was observed in the Uddanam area. During the dry season, elevated lead contents exceeding the threshold of 0.01 ppm, as specified by the Bureau of Indian Standards (BIS), were identified in 55% of the villages, while this incidence dropped to 105 in the wet season. However, ground water fluoride levels were found well below the BIS standard (1.5 ppm) in 95% of the villages (mean concentration was 0.54 ppm, SD = 0.40 ppm). Moreover, the presence of pesticides was not identified in any groundwater sample, while phthalates were detected in all samples [85]. Odisha is another region in India with a high prevalence of CKDu. Analysis of ground water samples in the area identified elevated fluoride levels above the WHO threshold (1.5 ppm) in 57% of drinking-water samples with an average fluoride content of 1.8 ppm (range: 0.58–4.95 ppm). Health risk assessment based on exposure pathways through ingestion and dermal contact identified a higher risk of non-carcinogenic health problems associated with high-fluoride exposure particularly for infants and children [86].

In addition to fluoride, hardness, heavy metals and metalloids, contamination of drinking water with certain agrochemical residues such as glyphosate and paraquat has also been reported in CKDu-impacted regions. For instance, a recent study analyzed 154 water samples collected from wells located in CKDu-endemic regions along with 50 water samples collected from wells in CKDu non-prevalent regions in Sri Lanka. Glyphosate contamination was detected in 44% of endemic wells and 8% of non-endemic wells, while fluoride was detected in 99% of endemic wells and 80% of non-endemic wells. According to logistic regression, the presence of elevated glyphosate, fluoride, hardness, and vanadium in wells was positively associated with CKDu prevalence. The co-occurrence of glyphosate with high hardness suggests potential complex formation between glyphosate and cations, which may increase renal toxicity, highlighting that fluoride exposure alone may not fully explain CKDu pathogenesis [46]. Although the contents of these nephrotoxic metals and metalloids in drinking-water samples in the CKDu-impacted regions are below the recommended thresholds, a rigorous assessment of exposure risks in these communities through paddy and other locally grown food crops is warranted for having better conclusions on the impact of these ions on the development and progression of kidney injury and subsequent CKDu susceptibilities.

When examining the geospatial distribution of these environmental risk factors, notable inconsistencies and heterogeneities emerge. For instance, in the regions where the drinking-water fluoride contents are high, water hardness remains relatively low, suggesting complex and non-linear exposure interactions that warrant detailed correlation and multivariate analyses. Additionally, the potential nephrotoxic effects of Cd, Pb, As, and Cr in combination, even at low concentrations, require further investigation. Importantly, geographic regions where multiple risk factors coincide (e.g., high fluoride, hardness, and trace metal mixtures) tend to correspond with CKDu hotspots, supporting the need for integrative studies on the synergistic and cumulative impacts of environmental exposures.

In summary, the evidence from Sri Lanka suggests a moderate to a strong association between fluoride exposure risk and CKDu prevalence. However, such relationships are not consistently observed in other CKDu-impacted regions such as India and Central America. Notably, several studies have reported the presence of nephrotoxic heavy metals and metalloids in environmental samples in CKDu-impacted regions and in biospecimen of CKDu patients at elevated levels compared to healthy subjects, reinforcing the need for multi-exposure risk assessments and integrative environmental–biological analyses to identify causal pathways and population-level susceptibilities.

##### Evidence from Autopsy and Renal Biopsy Studies

While environmental and geochemical analyses provide indirect evidence of fluoride and metal exposure in CKDu-endemic areas, histopathological and tissue-level investigations offer direct insights into the internal accumulation of these nephrotoxicants and their potential pathogenic roles. Autopsy and biopsy-based assessments are particularly valuable in understanding chronic exposures. A case–control study compared autopsy samples of bones from patients who died of CKDu in CKDu-impacted regions with samples from age-matched control subjects with no history of kidney disease who died in CKDu non-prevalent regions [87]. Although no significant differences in cadmium and mercury contents were observed between the two groups, the calcium-adjusted lead and fluoride concentrations in the bones of CKDu cases were significantly higher than those of controls [87]. It is important to note that progressive decline in glomerular filtration rate (GFR) is likely to occur with CKDu advancement. At sufficiently low GFR levels, renal fluoride clearance becomes impaired, since the kidneys represent the primary excretory pathway for fluoride. Consequently, fluoride accumulation in bones and its subsequent mobilization into circulation may exacerbate nephrotoxicity, potentially triggering a synergistic cascade of events which may lead to further deterioration in the GFR.

#### 3.2.4. Spatial Distribution of Fluoride and CKDu Prevalence

**Figure 6:**
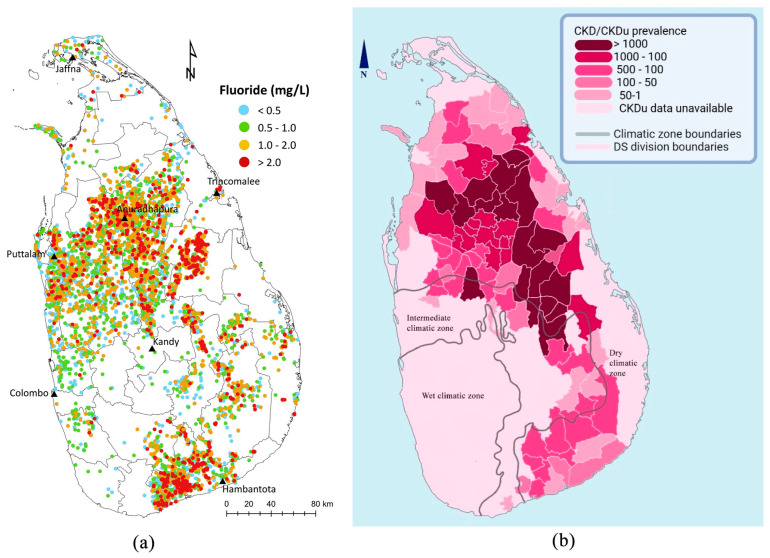
(**a**) Spatial distribution of fluoride in Sri Lanka, adapted from Gulegoda et al., 2022 [88], and (**b**) CKD/CKDu prevalence in Sri Lanka based on cases reported at the Divisional Secretariat level, as analyzed by Ranasinghe et al., 2019 [89].

Sri Lanka shows a marked variation in geogenic fluoride levels in drinking water depending on the region (Figure 6a) [88]. Considering the emerging evidence from Sri Lanka showing a potential correlation between fluoride exposure and CKDu, examination of spatial overlap of fluoride distribution and CKDu prevalence can be insightful (Figure 6b).

However, according to Gulegoda et al., there are some fluoride hotspots in the southern region of Sri Lanka, where CKDu is not prominently reported. Hence, these spatial data do not merely provide strong evidence for linking high drinking-water fluoride levels with CKDu incidence.

Moreover, geospatial distribution of drinking-water fluoride and CKDu epidemiology in India similarly does not demonstrate a consistent overlap. Figure 6a illustrates the geospatial distribution of drinking-water fluoride in India [2] along with the locations where clusters of CKDu cases have been identified in local communities [90].

**Figure 7:**
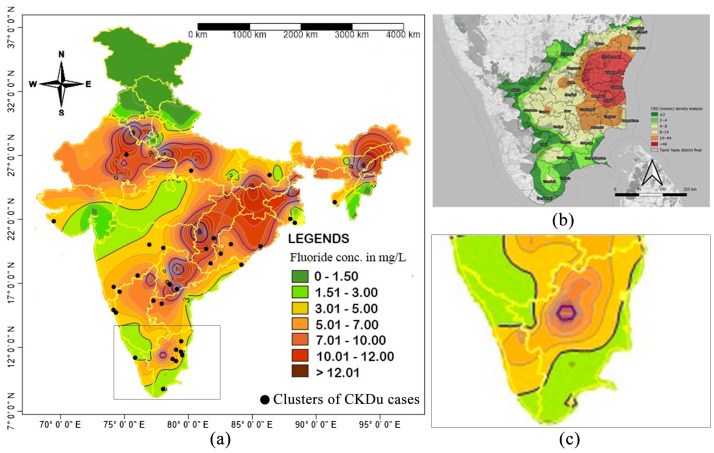
(**a**) Spatial distribution of drinking-water fluoride in India adapted from Mukherjee and Singh, 2018 [2]. Black dots in the map represent locations where clusters of CKDu cases have been reported within communities as presented by John et al., 2021 [90]; (**b**) Incidence of CKDu in the districts of Villupuram and Puducherry in southern India. Adapted from Parameswaran et al., 2020 [91]; (**c**) An overview of drinking-water fluoride distribution in the districts of Villupuram and Puducherry for comparison with CKDu incidence.

As Figure 6a implies, some of the CKDu clusters have been reported in regions with high fluoride contents while a majority of CKDu clusters occur in areas with relatively low fluoride concentrations. Moreover, Figure 7b depicts the epidemiology of CKDu in the districts of Villupuram and Puducherry in southern India [91]. Overlaying the CKDu hotspots in these districts with the corresponding geospatial fluoride distribution map (Figure 7c) reveals that the fluoride concentrations in these CKDu-impacted regions are relatively low [2].

Moreover, it is important to consider the communities residing in high-geogenic-fluoride content areas. For example, multiple studies have reported elevated drinking-water fluoride contents in the area. High-fluoride exposure is evidenced by the high prevalence of dental and skeletal fluorosis in communities. According to a meta-analysis by Demelash et al., 2019, the pooled mean ground water fluoride content was 6.03 ppm (95% CI; 4.72–7.72, *p* < 0.001), while the pooled overall prevalence of dental fluorosis was 28% (95% CI, 24, 32%, *p* < 0.001) [92]. Moreover, relatively high fluoride contents in drinking water are documented in multiple states in India including Rajasthan, Andhra Pradesh, Telangana, Tamil Nadu, Gujarat, and West Bengal [2]. Additionally, severe drinking-water-borne fluorosis has been documented in multiple regions in China. These include Heilongjiang, Jilin and Liaoning (Northeast Three Provinces), Shanxi and Tianjin (North China), Inner Mongolia, and Qinghai (Northwest China) [93]. The prevalence of fluorosis is definitive evidence of high-fluoride exposure in the communities. However, evidence of a high risk of kidney injury or incidence of CKDu is not consistently reported from these regions.

Taken together, geospatial analyses of fluoride distribution and CKDu case clusters suggest that regions with high natural fluoride content do not consistently overlap with areas reporting elevated CKDu prevalence. This discrepancy highlights the multifactorial and context-dependent nature of CKDu etiology. If excessive fluoride exposure were a primary driver of CKDu incidence and progression, one would expect to observe more frequent or pronounced CKDu clusters in regions such as parts of India where groundwater fluoride concentrations are exceptionally high. Several explanations may underlie these inconsistencies: (1) differences in co-exposures—such as varying levels of water hardness, heavy metals (Cd, Pb, As), or agrochemicals—that may modulate fluoride toxicity; (2) differences in genetic susceptibility, as population-level variations in detoxification pathways or renal transporter polymorphisms may alter vulnerability to nephrotoxins; (3) variations in occupational and lifestyle factors, such as heat exposure, hydration behavior, and dietary mineral intake, which could influence fluoride kinetics and renal burden; and (4) differences in diagnostic criteria, healthcare access, or surveillance intensity across countries.

Collectively, these factors underscore that fluoride exposure alone is insufficient to explain CKDu distribution patterns, and its pathogenic potential likely depends on the combined influence of environmental, physiological, occupational, and sociocultural determinants.

## 4. Conclusions

Population health studies in regions with elevated drinking-water fluoride levels underscore its association with adverse effects on both children and adults, notably evidenced by the prevalence of dental fluorosis. The high occurrence of fluorosis in areas such as India and China highlights the magnitude of fluoride exposure in these populations. Furthermore, biomarker-based studies show a correlation between high-fluoride exposure and kidney injury. However, in-depth studies with longitudinal data are required to understand the long-term kidney health impacts of fluoride exposure, particularly in communities residing in regions with higher geogenic fluoride contents.

Taken together, studies summarized in this review including those from Sri Lanka, suggest a possible association between fluoride exposure and CKDu incidence. Given the known nephrotoxic properties of fluoride, higher levels of this element, particularly in drinking water, could contribute to kidney damage and potentially influence disease onset or progression. However, considerable regional variations and diverse sources of fluoride present a challenge in determining potential mechanisms that may underlie the observed associations with CKDu incidence. Nonetheless, considerable regional heterogeneity, multiple fluoride sources, and methodological inconsistencies complicate the ability to draw definitive conclusions about fluoride’s role in CKDu pathogenesis. This complexity is further highlighted by the absence of consistent associations in regions such as India, where groundwater fluoride concentrations are high yet CKDu clusters are uncommon.

Studies summarized here show that chronic fluoride exposure risk may have preceded the emergence of CKDu. For example, the presence of dental fluorosis has been documented in multiple regions across the globe including in some of the CKDu-impacted areas for decades earlier than reports of CKDu. Importantly, almost all CKDu research and case reports emerged after 1990s and the prevalence of CKDu became increasingly evident after that in the currently known hotspots. If high-fluoride exposure alone is responsible for CKDu, regions with high dental fluorosis would overlap with global CKDu hotspots and would have become evident much earlier than the 1990s.

The lack of CKDu incidence in regions around the world with high risk of fluoride exposure indicates that while fluoride poses a risk to kidney health, other environmental triggers are also likely contributors to CKDu. Evidence points to anthropogenic and environmental contaminants as plausible contributors to noncommunicable diseases, including CKDu. It is plausible that CKDu is influenced by factors such as water hardness and fluoride, and contaminants such as heavy metals and agrochemicals that may collectively contribute to disease initiation and progression. These findings point toward a multifactorial etiology, where synergistic interactions between fluoride, water hardness, heavy metals, agrochemicals, and occupational heat stress may collectively contribute to CKDu pathogenesis.

Understanding these interactions requires more integrative and comprehensive research designs. Future studies should prioritize longitudinal cohort studies that track co-exposures and kidney outcomes over time, employ multi-omics approaches (transcriptomics, metabolomics, and proteomics) to identify molecular pathways affected by mixed exposures, and utilize high-resolution geospatial modeling to delineate environmental risk patterns with greater granularity. Incorporating systems toxicology and exposome frameworks could further elucidate how cumulative environmental burdens interact with genetic and physiological vulnerabilities to drive renal injury.

Despite the mixed evidence for an association between CKDu and fluoride globally, regular monitoring and better management of water supplies in CKDu-prevalent areas are crucial. For example, in Sri Lanka, according to the approximations by Ranasinghe et al., 2019, high fluoride levels in drinking water exceeding 1 ppm have affected 1.2 million (11.2%) inhabitants [94]. Moreover, 0.53 million (12%) of children below 12 years of age are living in high-fluoride areas (>1 ppm). In a global perspective, the estimated risk of high-fluoride exposure through drinking water (fluoride concentration above 1.5 ppm ranges between 63 and 330 million people (0.8–4.4% of global population) according to a recent analysis [18]. As implied by these statistics, high-fluoride exposure through drinking water has impacted a significant portion of the Sri Lankan population alone [94]. These statistics underscore the importance of sustained surveillance, risk communication, and targeted mitigation strategies to protect vulnerable populations. Ultimately, a coordinated global research effort that integrates epidemiology, environmental science, and molecular biology is crucial to unravel the true contribution of fluoride within the broader environmental landscape of CKDu.

## Data Availability

No new data were created or analyzed in this study. Data sharing is not applicable to this article.

## Supplementary Materials

The following supporting information can be downloaded at: https://www.mdpi.com/article/10.3390/toxics13110966/s1, Table S1: An overview on the community studies demonstrating potential association of fluoride exposure with kidney health.

## Abbreviations

The following abbreviations are used in this manuscript:

ACP	Acid phosphatase
AHR	Anti-hydroxyl radical
ASA	Anti-superoxide anion
BIS	Bureau of Indian Standards
BUN	Blood urea nitrogen
CAT	Catalase
CI	Confidence interval
cIMT	Carotid intima-media thickness
CKD	Chronic kidney disease
CKDu	Chronic kidney disease of uncertain etiology
CLU	Clusterin
eGFR	Estimated glomerular filtration rate
ET-1	Endothelin-1
FIN	Focal interstitial nephritis
GAP	Groundwater assessment platform
GND	Grama Niladhari division
GSH	Glutathione
GSH-Px	Glutathione peroxidase
ICAM-1	Intercellular adhesion molecule 1
KIM-1	Kidney injury molecule
LDH	Lactate dehydrogenase
NAG	N-acetyl-beta-D-glucosaminidase
RBP	Retinol binding protein
ROS	Reactive oxygen species
SOD	Superoxide dismutase
UA	Uric acid
VCAM-1	Vascular cell adhesion molecule 1
WHO	World Health Organization
