# Supplementary material for: Is Fluoride the Culprit? Revisiting Evidence on Environmental Origins of Chronic Kidney Disease of Uncertain Etiology (CKDu): A Narrative Review
Source: Toxics. 2025 Nov 10;13(11):966. doi: 10.3390/toxics13110966 (PMC12656354; doi:10.3390/toxics13110966)
Supplement: Supplementary file 1 [file toxics-13-00966-s001.zip › toxics-3905742-supplementary.pdf]

Supplementary Table S1: An overview on the community studies demonstrating potential association of fluoride exposure with kidney health.

Study	Study setting and participants	Fluoride Exposure statistics	Key findings
Khandare et al., 2017 [49]	A Case control study with 824 school students (8-15 years of age) in fluorosis-affected region in Doda district, Jammu and Kashmir, India.	Control UF: 1.91 ± 0.64 ppm*<br>Cases UF: 3.28 ± 1.71 ppm*	Significantly increased serum creatinine and declined eGFR in students with dental fluorosis compared to the controls.
Quadri, et al., 2018 [50]	A Case control study with 32 children diagnosed for nephrotic syndrome with high urinary fluoride, and 33 age matched healthy controls (4-12 years of age) in India	Control UF: 0.56 ± 0.15 ppm*<br>Cases UF: 4.01 ± 1.83 ppm*	Electron microscopic observations in renal biopsies identified subcellular ultrastructural changes and tubular epithelial damage in the cases.
Kumar, et al., 2017 [51]	A cross-sectional study with 842children (8-15 years of age) from fluorosis-affected regions (Jhajjar, Dadanpur and Dariyapur) in Inda.	WF: 1.63 - 3.33 ppm†<br>UF: 0.05 – 2.80 ppm†<br>SF: 0.07 – 1.05 pm†<br>DF: 36.84% -94.63%	A significant positive correlation of the prevalence of dental fluorosis with fluoride contents in drinking water, urine, and serum.
Jiménez-Córdova et al., 2018 [53]	A cross-sectional study with 239 adults (18–77 years of age) from three communities in Chihuahua, Mexico.	WF: 1.5 (0.19-1.8) ppm‡<br>UF: 2.0 (1.1- 3.5) ppm‡	Multiple linear regression models identified a positive association of urinary fluoride level with multiple urinary markers including albumin, cystatin-C, KIM-1, osteopontin, and eGFR.
Jiménez-Córdova, et al., 2019 [54]	A cross-sectional study with 374 school children (5-12 years of age) in municipalities of Hidalgo del Parral and Aldama in Chihuahua in México	DF: 1.93 (0.3–2.1) ppm‡<br>UF: 2.7 (2.0–3.6) ppm)‡<br>DF: 41.7%	Multiple linear regression models identified positive associations of urinary Fluoride with eGFR, VCAM-1, ICAM-1, and cIMT, and negative associations with urinary and serum Cystatin-C.

Malin et al., 2019 [55]	A cross-sectional study with adolescents aged 12–19 years in National Health and Nutrition Examination Survey (NHANES) in USA	DF: 1.93 (0.3–2.1) ppm• UF: 2.7 (2.0–3.6) ppm•	1 µmol/L increase in plasma fluoride associated with a 10.36 mL/min/1.73 m2 lower eGFR.
Wu, et al., 2021 [56]	A cross-sectional study with 1,070 adults (mean age 58 years) in four villages in Wenshui County, Shanxi Province in China	UF: 1.62 ppm**	1 ppm increment of urinary fluoride was associated with 1.583 U/L increase in urinary NAG and 0.199 mmol/L increase in serum urea
Lavanya, et al., 2025 [57]	A cross-sectional study with adults (21-60 years of age) in endemic fluoride villages of the YSR Kadapa district in India.	Mean WF: 1.5 – 4.1 ppm†	a strong positive correlation of urinary fluoride with serum creatinine and blood urea nitrogen. Drinking water fluoride showed positive correlation with serum creatinine and blood urea nitrogen.
**Abbreviations:** DF: dental fluorosis incidence rate, UF :Urine fluoride level, SF: Serum fluoride level, WF: drinking water fluoride level **Indications:** * Mean±Standard Deviation, # mean (Standard error), † Range, ‡ Median (Interquartile Range), • mean (Interquartile Range), **mean			
